# Online Collaborative Documents as Media Logic: The Mediatization of Risk Response in the Post-pandemic Era

**DOI:** 10.3389/fpsyg.2022.892569

**Published:** 2022-06-23

**Authors:** Hao Jiang, Yi Zhang, Wodong Guo, Wei Cheng, Jian Peng

**Affiliations:** ^1^Institute of Journalism and Communication, Sichuan Academy of Social Sciences, Chengdu, China; ^2^School of Communication and Design, Sun Yat-sen University, Guangzhou, China

**Keywords:** online collaborative documents, mediatization, technological affordances, risk communication, affective publics

## Abstract

Online collaborative documents (OCDs) have previously been the focus of office efficiency, but today they can be a special approach to risk response in public health and natural disaster situations. Studying the mediatization of the risk response by OCDs can help us understand the interaction between digital technologies, online users, and emotions in a post-pandemic world. A mixed-method design involving online ethnography and focus groups was employed to discuss OCD performance during the 2021 Henan flood. The empirical results indicate that four dimensions of technological affordances (i.e., editability, accessibility, activability, and normability) connected the functional features of the digital platform with users’ potential actions. Risk communication as a contextual element of media exposure and discursive practice provided a participatory and constructive framework for users’ gathering. Therefore, affective ties including anxiety, fear, and encouragement supported the affective publics’ mass deliberation and social mobilization. These findings provide an institutional lens for mediatization research to view OCD as media logic and reveal some methods that can be referred to for risk management and humanistic concerns globally.

## Introduction

During the COVID-19 pandemic, people have increasingly relied on various media to search for information about COVID-19, access news stories (Yue and Yang, 2021), maintain social contact (Shearer and Mitchell, 2021), and even seek entertainment to maintain their mental health (Yang and Ma, 2020) due to policy restrictions such as stay-at-home orders, mandatory quarantines, and social distancing. Media was previously merely a supplement to face-to-face communication, but now it is replacing offline interactions (Choi and Choung, 2021). The role of media technology in human society has never been so complex, and it has significantly “mediated” the global publics’ daily lives. Thus, reconceptualizing human interactions with media technologies is now a challenge that requires exploration and the discussion of certain forms of digital platforms.

In the wake of disasters, scandals, and other unexpected events, unique forms of social behavior and organization emerge to support information communication, responses, and the construction of meaning. On Mainland China, an online collaborative document (OCD) gained the public’s attention for its excellent performance in terms of risk response during the 2021 Henan flood. (To view the original link, please click https://docs.qq.com/sheet/DUG9pRWRsSlRyeHVn) On July 20, 2021, the Henan Provincial Meteorological Department recorded a single-day precipitation level of 449 mm in Zhengzhou, which was 288.74% of the historical average precipitation for this month. Water levels on the surface rose rapidly within hours, destroying power lines, energy, roads, communications, and sanitation facilities and affecting production and service sectors as well as public infrastructure. According to the State Council’s investigation report (Disaster Investigation Team of State Council, 2022), the flood disaster caused approximately 40.9 billion yuan in damage, 380 deaths and disappearances, and the evacuation of approximately 100,000 people. However, the government departments’ emergency response in the disaster’s early stages was not timely enough, so urban people actively self-organized rescue operations among their families and communities. Several agencies also initiated rescue programs, including information exchange, medical advice, alert systems, and evacuation guides. One OCD was converted from an office tool to a collaborative medium by citizens. The content editing, discussion, and sharing of the public issue of mutual aid and disaster relief were widely distributed (19 million views and 250,000 words on the OCD and 300 million views from related topics on social media) and constructively improved the efficiency of the risk communication and disaster relief management.

Online collaborative documents are efficient tools that apply a multi-person collaborative authoring model to text editing. The technology is similar to Wikipedia in terms of stimulating the potential for group intelligence. The existing major products are Microsoft Office for the web, Google Docs, Tencent Docs, etc. Are OCDs a digital media in the universal sense? Obviously, they are constructed with digital technology and have a certain potential as they can be used by humans to store and transmit specific information. A few interesting contributions aiming to discuss OCDs’ educational applications have emerged in recent years, such as Khalil (2018) and Woodrich and Fan (2017), who mention Google Docs as a useful tool for collaborative learning that is enjoyed by students. However, the existing research focuses more on the computer science significance of OCDs—such as the algorithmic architectures used for flexible encryption (Lv et al., 2016), content integration, and the implementation of complex functions such as real-time recall and damage identification (Wu et al., 2010). The information-mediating properties of OCDs, including their extensive relationships to social behavior or psychology, have not been sufficiently discussed. The gap in the relevant research is largely due to the lack of empirical cases, making it difficult for researchers to conceptualize the communication phenomenon. The use case in the 2021 Henan flood provides a unique window into events to understand the nature of the OCD medium and the social interactions it supports.

## Literature Review

### Mediatization and Media Logic

Mediatization is a concept with great potential for constituting a space for an integrated understanding of media-related social practices. In contrast to other research paradigms, mediatization theory invites systematic empirical research on various sub-processes to collectively provide a complex picture of how culture and everyday life evolve in an era of media saturation (Ekström et al., 2016). Through the work of Asp (1990), Hjarvard (2008), and Couldry and Hepp (2013), mediatization is defined as aiming to reveal how the presence of media ensures that interactions between actors take place. In contrast to the “mediated” model, the social interactions explained by mediatization are represented as follows:

*actors—media as an actor (media logic exerts effects)—actors → the mediatization of social practices*

The consolidation of mediatization theory has supported the activation of empirical studies in recent years—such as the mediatization of culture and society (Al-Zaman, 2022; Shi, 2022), politics (Ragragio, 2021), and journalism (Blassnig and Esser, 2022)—as well as the material dimensions of mediatization processes (Kannengießer and McCurdy, 2021). As Hepp (2012) asserts, media should not be understood in this model of social interaction only as an actor that leads to causality but as a force that supports and molds action. Meanwhile, the ambiguity of this theoretical framework resides in how to clearly define what it claims to be *media logic*. Media logic began with the journalism studies of Altheide and Snow (1979), who understood media as a communication form that possesses its own logic; thus, media logic means the form in which events and ideas are interpreted and presented. In addition, Asp (1990) first associated media logic with the study of mediatization, arguing that media logic is the system of norms in the process of media production. Apparently, these grand narratives seem to describe media logic as an undifferentiated concept, into which all the studies related to media effects can be placed (Deacon and Stanyer, 2014). Thus, critiques of media logic have been common in recent discussions of mediatization theory (e.g., Couldry, 2008, 2012; Lundby, 2009). One of the concerns is to avoid the indiscriminate use of media logic when conceptualizing specific communication phenomena or invoking other theories, such as materiality, media affordances, social construction, and actor–network theory. Therefore, media logic is more akin to a rhetorical lens that invites us to scrutinize the technological features of the media as well as the contextualized social institutions and cultures. We understand the process of risk response reshaped by OCDs as follows:

*actors (online users)—OCDs as media actors (key media logic: human–technology interaction, the social context of media exposure)—actors (online users) → the networked publics and social practice*

In this mediatization theoretical framework, we focus on three sub-processes to discuss the potential of OCDs to reshape social interactions: human–technology interaction, social context, and the networked publics and social practice.

### Human–Technology Interaction

Human–technology interaction is an active multidisciplinary field, involving research in psychology, communication, sociology, and philosophy. However, as Thüring and Mahlke (2007) worried, previous research has almost exclusively concentrated on the aspects of usefulness and usability. Technological affordance is perhaps a potential explanatory framework for examining the connection between actors and the technological environments in which they are located (Gaver, 1991). Affordance theory originated in perceptual psychology, where Gibson (1979) interpreted it as the possibility of action available in the environment. It aroused researchers’ interest and has since become a fundamental concept in ecopsychology (Bickhard and Richie, 1983; Stoffregen, 2000). Affordances in the literature about telecommunications are usually used to explain the characteristics, features, or hints of technology (Aakhus, 2007; Leonardi, 2011). Among the applications of media research, smartphones (Schrock, 2015; Mascheroni and Vincent, 2016; Jin and Cai, 2021) and social media platforms have been extensively discussed (Kaun and Stiernstedt, 2014; Ilten, 2015). Notably, Evans et al. (2017) proposed a set of threshold criteria to distinguish between “characteristics” and “affordances,” providing a constructive starting point for researchers to make inductive and conceptual deductions about phenomena. Those studies have shown that affordance is often used to describe the connecting relationship between subject and object, and the plurality of branching concepts is derived from the complexity of the object. For this reason, technological affordance applies to our discussion of human–technology interaction, which suggests a relational approach to understanding how people interact with technology (Leonardi, 2013) and aims to reconcile the contradiction between technological determinism and social constructionism.

Moreover, as Nagy and Neff (2015) noted, when the concept of affordances migrated to the field of communication technology and media studies, researchers continued to emphasize that affordances are environmental and material while ignoring or denying what is perceptual and imaginary. This context deviated from the original theoretical intent. Since the operation and propagation of OCDs are internet mediated, we expect that the specific characteristics and capabilities of the technology platform have an impact on the interaction mechanism. Nevertheless, technology has become more than a tool in human society over the digital era; it is the material basis for triggering a series of consequences of social action or changes in institutional structures. Technological affordances “create possibilities” for “everyday life” in a social sense (Wellman et al., 2003), and they have changed communication practices or habits (Schrock, 2015). Thus, the materiality of technologies is shaped in part by their sociality, a relational entity that bears traces of human activity (Turkle, 2004), including even psychological factors such as imagination, anxiety, and anticipation. Analysis of Belli (2021) of how socio-material networks’ affordances enable online collaboration supports this proposition. In a word, affordances can cause imagination, action, and other consequences, but they are not themselves the consequences. Therefore, this paper understands technological affordances as the potential actions that arise from the given technological features and forms, and poses the following research question:

RQ1: What are the technological affordances of OCDs?

### Media Exposure Context

On July 20, 2021, rare heavy rains formed floods that affected some communities in Zhengzhou. At 21:00, the first version of the examined OCD was created by a college student living in Zhengzhou, in the form of a spreadsheet named “Information on persons to be rescued.” After 5 min, two volunteers were added to the second version as collaborative managers to work on the information layout and checks. Within 2 h, the OCD was inundated with hundreds of user-initiated requests for help, such as, “A family of three trapped on subway line 5,” and “We urgently need a lifeboat to transfer the injured.” At 00:00 on the 21st, the OCD had a new sheet added, “Supportable,” showing some evacuation locations such as libraries, gyms, and cafes. Apparently, the OCD was being used as a form of risk communication in a crisis. Risk communication is defined by the U.S. National Academy of Sciences as a systematic, structured, scientifically based method for communicating effectively in high-concern, high-stress environments. These environments include any situation in which individuals or groups perceive a threat to their health, safety, or environment (Covello, 1998). This research perspective is often used to analyze how social actors use technological systems such as smartphones, online wikis, and social media to organize special responses, process and deliver information, and provide social support (Shklovski et al., 2008; Starbird et al., 2010) so the social actors can motivate the public to rationally perceive the disaster risks and take appropriate response measures to reduce risk hazards and maximize the protection of their property and health (Glik, 2007).

Inadequate communication in a crisis can result in negative emotions and stressful behaviors, so effective risk communication is a fundamental measure of the health emergency response (Reynolds and Quinn, 2008). Many studies during the COVID-19 pandemic have also shown that risk communication is now a major factor in public-initiated media exposure (Melki et al., 2020; Chu et al., 2022) and a significant way of regulating mental health (Wang et al., 2021). While classic research has often viewed government agencies and journalism as the core sectors of risk communication, digital technology now presents additional opportunities. The California wildfires are a good empirical case. Local residents took photos of the fire and updated them in real time on Twitter, so firefighters learned of the fire’s movements before reporters arrived on the scene (Sutton et al., 2008). Risk communication is coupled with digital media, and crisis response activities, both onsite and online, are becoming increasingly simultaneous and intertwined (Palen et al., 2007).

In addition, several studies have shown that building trust is an important factor in the public’s acceptance of information when facing threats and information overload (Kiousis, 2001). The level of media trust depends on media types, frequency of exposure, and user experiences (Tsfati and Cappella, 2003; Johnson and Kaye, 2009). Thus, from a media constructivist perspective, trust arises from the discourse and social interaction between media and users. For example, during a crisis, when people are aware of threats, they tend to seek authoritative information from mass media that emphasize expert sources (Loges, 1994; Lowrey, 2004), but they can also selectively access journalistic disaster coverage to prevent post-traumatic stress disorder (PTSD; Romer and Jamieson, 2021). Meanwhile, social media is used more often to express emotions (Denemark and Chubb, 2016). In many cases, the relationship between media use and media trust does not follow a perfectly rational model, as Tsfati and Cappella (2003) argued, in which the contents of the media messages constitute social attitudes and informs social behavior. Public opinion is seen by most risk researchers as a mass phenomenon that readily responds to information inputs and that tends to evade the psychosocial aspects of risk communication (Wilkinson, 2010). Therefore, relevant research should be conducted and not merely in the form of cross-sectional surveys but also by paying attention to more affective narratives.

The chosen risk communication strategy is closely linked to the type and progression of crisis events. In many classic cases of risk communication, persistent risk threats—such as health issues including SARS, COVID-19, HIV, and GMOs—focused more on the risk perception aspect in attempts to motivate citizens to adopt public policies and prevention recommendations and to influence their health behaviors (Slovic, 1987; Lee-Baggley et al., 2004; Brewer et al., 2007). In contrast, sudden crisis events such as the California wildfires and terrorist attacks fall under the topics of disaster response and post-disaster psychological support (Rogers et al., 2007; Sutton et al., 2008). Thus, this study avoids emphasizing the risk perception aspect. Because the citizens of Zhengzhou were facing an ongoing existential crisis at that time, the work of risk communication was to discuss relief strategies and care for mental health. During the flood, the central and Henan provincial governments continued to release weather conditions, rescue progress, and casualty figures. Journalism was also running feature stories. In addition, social media continued to contribute citizen journalism and emotional narratives. Tens of thousands of people simultaneously posted requests for help or made personal donations at the OCD. Other netizens discussed the incident intensely, and rumors were widespread. Therefore, this paper poses the following research questions:

RQ2: (a) How did the flood-affected groups use media for the risk response? (b) What were the characteristics of the OCD-mediated risk communication compared to other forms of digital media?

### Networked Publics and Social Practice

Networked publics are usually understood as a contested and messy term with multiple meanings used across different disciplines (Boyd, 2010). Similar to the concerns of mediatization studies, contributions on networked publics highlight that “publics can be reactors, (re)makers and (re)distributors, engaging in shared culture and knowledge through discourse and social exchange as well as through acts of media reception” (Ito, 2008). Thus, the mediatization of social practices should try to move toward the space where interactions occur between people, technology, and the imagined collective. These processes lead to some uncertain affective results, such as the erosion of community vitality and social cohesion that occur under same-day-delivery online shopping conditions (Xi et al., 2021). To be sure, digital media are not only containers for citizens’ emotional expression but also active creators of socio-emotional space (Giaxoglou, 2015; Sumartojo et al., 2016). When some political and cultural issues become mediatized, the affective flow becomes highly relevant (Hepp, 2012; Deacon and Stanyer, 2014; Savaş, 2019). This phenomenon or trend of social publics engaging in online activities around emotional ties is conceptualized by Papacharissi (2015, p. 126) as affective publics: a networked public formation of connection—identification—disconnection that is mobilized through expressions of sentiment.

The magnitude of the impact of flood events is usually quantified in climate and economic terms. However, in the aftermath of a flood response, concern for the victims and the disaster’s long-term effects urgently requires an emotional component (White et al., 2010). Emotion is likened by some researchers to Higgs Boson’s particle in the social sciences. Among efforts to apply the “affective turn” of global social science research to the interpretation of media technology and user activity, Papacharissi’s research makes a clear and valuable contribution. In her book *Affective publics: Sentiment, technology, and politics*, she theorized the structure of the affective public: First, the public in the internet era is primarily an affective community. They gather around media and platforms, invite affective coordination, support affective investment, and spread affective expression. Second, based on understanding of Boyd (2010) of networked publics, affective publics are considered publics transformed by networked technologies. Technological affordances are thus an important driver for the formation of affective publics, and shareability is summarized as the core affordance characteristic that either invites or discourages a particular type of social activity. Third, the sharing of sentiments on social media platforms is not limited to the intragroup but interacts with the larger social context and institutional environment. The media do not directly facilitate the negotiation of collective identities, so networked publics need specific political and cultural discourses. In psychological experiments, sentiment can be measured directly by a variety of methods, but communication studies require specific mediators. Affective publics often leave distinct digital footprints in unique ways, such as various forms of media texts (Dean, 2010; Papacharissi, 2016), so this paper poses the following research question:

RQ3: (a) What were the main affective flows active in the OCD? (b) How do users socially mobilize around affective ties?

## Materials and Methods

As an ideal example of executing in an online interactive space, this OCD preserved the memories from the media technologies, texts, and experiences generated by the public’s interactions with specific digital media during the flood. The aim of this paper is to reveal the potential of OCD technology to reshape risk response through media logic. A mixed-method design involving online ethnography and focus groups was employed to discuss the mediatization of the risk response.

### Participants

Inclusion criteria were living in Zhengzhou during the 2021 flood and being at least 18 years of age. A focus group containing 10 people was interviewed in a semi-structured manner during late January 2022. They all gave their consent for the interviews to be collected, preserved, and processed by the researchers. Table 1 reports the demographic information for the focus group participants.

**Table 1:** Demographic information for focus groups.

No.	Gender	Age	Identities in OCD	Occupations
A1	Male	50	Help seeker	Farmer
A2	Male	43	Help seeker	Company Staff
A3	Female	23	Help seeker	University Student
A4	Male	37	Help seeker	Company Staff
A5	Female	35	Help seeker	Company Staff
A6	Male	19	Help seeker	University Student
B1	Male	23	Volunteered maintainer	Company Staff
B2	Female	26	Volunteered maintainer	University Student
B3	Female	29	Volunteered maintainer	Company Staff
C1	Male	51	Official government	Civil servant

### Research Design

Before discussing the OCD’s performance in a targeted manner, it is necessary to understand the media usage of Zhengzhou citizens during the flood period. Thus, online questionnaires were placed on social networks by a research partner in Zhengzhou in January 2022. Participants were informed of the study’s purpose and the privacy policy before completing the questionnaire. The researchers distributed 500 questionnaires, and after eliminating invalid data (the respondent did not live in Zhengzhou or was below 18 years old), a valid sample of 420 was collected. The measurement items covered the frequency of media usage, the motivation for the media exposure, media trust, and OCD exposure. All results of the descriptive statistics are illustrated in Supplementary Material.

Similar to most media technology-oriented affordances studies (e.g., Majchrzak et al., 2013; Kaun and Stiernstedt, 2014), we did not attempt to extensively map user behaviors and content generation but rather to discover the interface between technological potential and social actions. We first approached the technological materiality of the OCD, roaming contextually through the functional support and content creation aspects of the platform to inform the interviews on the issues related to the users’ experiences on media usage and technology adaptation. In the focus group, the participants were first asked a few targeted and fixed questions that prompted them to share their media usage experiences during the flood. We also asked specific questions about the underlying goals, experiences, and practices associated with OCD with the document maintainers who had been granted higher privileges by the creators. To extract rich data, personalized secondary questions were created for further in-depth study. Beyond the construction process, each participant gradually and collectively built a shared story by sharing individual stories. Supplementary Material contains the main questions.

For the affective characteristics of the OCD, media texts from 10,589 posts totaling approximately 74,300 words were selected as the sample for sentiment analysis. We used the Jieba Chinese word splitter running in the Python environment to split and count the text. In addition, the LIWC-22 Chinese dictionary was used to identify and label the text with affective words, resulting in the four emotion dimensions of “positive,” “sad,” “angry,” and “anxious.” Then, we added and adjusted some custom words based on the specific text of the case study, and selected the one with the most hits among the four types as the affect category of a text; those without hits were marked as “others.”

## Technological Affordances

### Editability

As an efficiency tool originally applied to mental work, OCDs enable the writing and storing of multimedia content such as text, images, video, and audio in online multi-person collaborations. In terms of the technological materiality, OCDs are fixed entities in itself. They are like blank sheets of paper that continually acquire new meaning through a series of users’ editorial actions. The technology platform that supports OCD operations enables users to freely add, review, recall, evaluate, and modify multimedia information in a participatory manner within the scope of the permissions they are allowed. Two of the project’s early managers interviewed, when asked why they chose an OCD as the primary digital media platform for information storage and communication, described it this way:

*I had almost no hesitation in thinking it would achieve our goals. Because we use it regularly to register personal information in workgroups or to record brilliant inspiration for brainstorming. OCD seems easy, reliable, and efficient … The great user experience in the past does not lie. (Interviewee B3)*

*The moment I visited the OCD, it was as if I was thrown into a scene where I was performing emergency work. While this is true in reality, I want to emphasize that it was only after interacting with the OCD that I realized it fit perfectly. (Interviewee B2)*

Obviously, the analysis of affordances relies on the media usage experience. OCD is very similar to Wikipedia in terms of shaping a participatory information environment. As technological mediators of human activities centrally organized around shared practical understandings, both platforms aim to significantly advance the wisdom of groups through the active participation of individuals. Compared to other digital technology systems, such as television or social media, OCDs appears to be better suited to support *ad hoc* organizations in documenting breaking events and can be considered a paradigm of timeliness and reliability. OCD tasks may seem highly interdependent and incur high negotiation costs between editorial groups. However, unlike many other cases of collective intelligence, the scalability of OCDs allows users to strategically adapt the technological conditions of the platform to achieve the stated goals. Scalability represents highly flexible and user-friendly technological features, such as automatic saving without manual confirmation and real-time recall. Furthermore, each OCD has history pages stored on the cloud server, where users can access all previous versions. However, the time and attention required to do so on traditional digital technology systems such as WeChat, WhatsApp, or Twitter can be fatal in an emergency.

### Accessibility

Emergency disasters can damage digital infrastructures such as communication base stations and wires to a varying degree. Most digital technology systems require a constant supply of electricity and the internet, but smartphones allow people to access and post information during power outages, if battery support continues. Thus, mobile internet has now become the primary way of maintaining communication between victims, experts, volunteers, and government agencies in crisis (Bunce et al., 2012). Social media apps, as a way of accessing and sharing information, ensured their importance as a warning system and by providing relief to those affected by the floods in Zhengzhou. However, evaluating the role of social media in terms of efficiency, they continued to lag somewhat in terms of messaging. Based on the respondents’ media usage experiences, we learned that if a family faced a flood threat who could not reach 110 (similar to 911 in the United States) had several ways available of seeking help through Weibo (the most popular social media platform for strangers on Mainland China):

*1. Find the contact information of the relevant rescue team in the search bar and try to report your location accurately.*

*2. Choose the correct tag (e.g., #Mutual help in the Zhengzhou flood) and post the message. After that, wait for a reply.*

Obviously, obtaining official relief in the early stages of the flood was difficult, which was exactly why Zhengzhou citizens were actively engaged in mutual aid initiatives through the OCD. As software developers claim, OCDs’ multi-scene compatibility supports their core competencies. A series of streamlined features allow OCDs to run on mobile apps, web browsers, and even as a built-in component of some social media platforms. For those in desperate situations, OCDs can be used without loading images or videos; they do not require a high level of user media literacy or mobile hardware performance; and they consume very little cellular network traffic, battery power, and user attention.

Additionally, OCDs are inherently open access, they support decentralized operations, and they are richly scalable unless controls are artificially modified or access is restricted. Taking as an example Google Docs, various application programming interfaces (APIs) realize a seamless connection between users and related expansion features. Examples include automatically recognizing and filling in phone numbers from contacts or displaying your location information *via* Google Maps with one click. While Google services are restricted on Mainland China, fortunately, Tencent Docs, the case for this study, has similar scalability, can be shared unhindered, and runs smoothly on social media platforms. The Tencent Maps API was accessed for users to automatically mark and upload flooded, electrical leakage, collapse and evacuation spots in flooded areas. The home page was a navigational sheet that aggregated all of the OCD’s functions, including internal hyperlinks that took the users to sub-pages about seeking support, danger area warnings, psychological counseling, public health recommendations, charitable donations, etc. Furthermore, OCDs are highly scalable on the server side. Interviewee B1 told us how the volunteer team negotiated with the service provider to accommodate more users. Overall, OCDs’ accessibility means more opportunities for users’ media exposure.

### Activatability

Online collaborative document is not a “meta-media,” which would need to operate in an existing polymedia environment. During the flood, the OCD was frequently viewed on social media such as WeChat groups and Weibo, with a significant attachability. We expect that social media can occasionally be used for public goals or to advance public interests (Baym and Boyd, 2012; Poell and Van Dijck, 2016), which helps to activate and maintain potential relationships and is essential for mobilizing online publics (Karatzogianni and Kuntsman, 2012). In addition, social networks and their technological support significantly influence the publics’ emotional experience, either as an outlet for expressing sentiments or as a tool to reinforce existing ones (Choi and Toma, 2014). The OCD activated social connections through the social networks to which it was attached, including information-sharing and emotional contagion. However, the online activity did not guarantee impact; it merely gave momentum to action and facilitated potential engagement, including psychological factors. Message texts were posted by users in the specified location in the OCD and occupied one or more cells. This editorial approach created opportunities for others to participate in conversations, and other users could show their support by commenting. In addition, the OCD allowed text messages to have rich formatting characteristics, such as customizable fonts, sizes, and background colors. Impressively, we found a message in the OCD from an out-of-town visitor describing himself besieged at a railroad station and complaining of bad luck, but cells around it were filled with others with pink background colors, heart-shaped emojis, and encouraging comments. The discursive practice of these affective expressions seems incredible. In contrast, while other social media platforms such as WeChat and Weibo also promoted spontaneous interaction and information-sharing, they were slightly more mediocre at activating creative expressions of sentiment.

Online collaborative documents support users in expanding weak social ties rather than in strengthening existing strong social ties. This activatability has led OCD to become a platform better capable of activating social mobilization. Activatability is not the same as collaboratability. Evans et al. (2017) similarly suggested that researchers should be cautious in assessing the threshold for using the “collaborative affordance” as a branching concept. Collaboration is a relational act and can be used to describe a practical consequence of actors, rather than a property of the technology itself. Only when OCDs interact with online users and activate their potential actions does collaboration become possible.

### Normability

Coordination in unstable environments such as disaster responses or emergency medical care situations requires a high degree of caution and interrelated knowledge integration and information-processing actions (Brown and Eisenhardt, 1997). The press and social media were committed to contributing to the information flow during the Henan flood and influenced social actions to some extent. However, the public’s interactions with journalism were limited because in times of crisis they looked forward to seeing dedicated rescue teams rather than journalists. Similarly, distress messages were hardly guaranteed to be widely read and effectively responded to in the fragmented information environment of social media, although some specific hashtags were set by Weibo users. As Poell and Van Dijck (2016) note, the public moment will certainly be temporary, as social media constantly tries to link user action trends to commercial advertising. Unlike the mass communication sector, social media, or Wikipedia, which have clear user agreements and community norms, OCDs do not prominently inform each user of the rules, etiquette, and guidelines of this digital space. Instead, the technology facilitates or restricts users’ certain potential actions through its technological conditions.

*What sticks in my mind is that in a panic I was trying to edit the text I had already posted, but accidentally selected others’ one and pressed the delete button. The system informed me that the delete operation was invalid, and then I had known that each non-managers editing privileges were restricted to only being able to manipulate what they had posted. (Interviewee A1)*

*It was reassuring that all the information within OCD seemed to be themed around the floods and able to contribute to the relief process. I did not find any commercial ads during my browsing, while it was appearing frequently on the Tik Tok app or TV channels. (Interviewee A3)*

*The OCD creators were wise to choose spreadsheets over Word and slides. Cluttered information could be automatically categorized and shown in various cells. (Interviewee A6)*

Obviously, the information flow in the OCD was dense and pure, as it avoided the distraction of commercial advertising and entertainment content. Many people had never worked together previously on a public affair through online collaboration and may never do so again. However, they had some autonomy and imagination in shaping the digital technological infrastructure to achieve their own aims. The users also understood a range of cues about appropriate or acceptable behavior based on the interactive experiences, and then adapted more or less to the norms of the digital space. The normative affordance of the OCD supported the construction of an institutionalized group coordination mechanism, which is the main reason this empirical case took place on this media platform rather than on other available ones.

## Risk Communication

### Participatory Risk Information Release

Geographic information systems (GIS) and internet technologies have increased the validity of perceived consequences regarding the population and improved the publics’ ability to make decisions in risk response situations (Brown et al., 2017). Nevertheless, digital participation is often seen as an elite technology that requires a certain level of intellectual literacy and excludes some people from the information release process (White et al., 2010). In contrast to those previous studies, we found that OCDs’ four dimensions of technological affordances facilitated public participation in risk communication and simplified the consultation process among stakeholders such as victims, volunteers, and government agencies. Through participatory knowledge-sharing and experience exchanges, basic precautions, known risk areas, evacuation routes, and available dietary supplies were identified. Moreover, the release process generated reliable, quantitative, and easy-to-use material that could be observed and analyzed by researchers. Participatory information release treats meaning and experience as emergent, and as Deetz and Brown (2004) argued, meaning is initially formed in the relationship between goal-directed activity and what is yet to be decided in the world. Individuals may be the gateway to perceiving new cues and discussing collective decisions, which leads to productive participation. Because personal narratives are sometimes more relevant to the interests of the audience than are factual truth and data, and are more likely to raise concerns about risk, they can quickly gain attention. We believe that democratized, broad-based participatory action can improve decision-making; increase acceptance, ownership, and commitment to desired programs; and incorporate lessons learned into new programs and developments.

This information-sharing network had no fixed center and its structure evolved dynamically. The actors enhanced their positions in this network by absorbing more information and processing it effectively. If they performed poorly, others took over their tasks. For example, People’s Daily and Xinhua News Agency also appeared on the OCD’s homepage, but their position did not depend on their inherent social capital and information power. Instead, it was seen by the other actors as a trusted source contributing to the environmental perception. Therefore, the information release action was based on a practical contribution rather than cultural capital or power confrontation, which led to coordination mechanisms that emphasized participation, reciprocity, and reputation. Further, open, democratic as well as sustainable risk information release activities could even positively lead to mindfulness in the cognitive psychological sense, such as minimizing the level of distraction and leading to adjusting the metacognition of risk information (Kudesia, 2019). As a result, the participants acquired a collective subjectivity organized around the right to live in the practice of responding to a risk crisis.

### Constructive Issue-Oriented Communication

Many risks are difficult to avoid or manage because of problems in predicting or quantifying them. Moreover, the process of media coverage is not merely a simple reproduction of events but a constant process of interpreting and transforming the meaning of reality and possibly constructing new risks. In the 2021 Henan flood, the emergency response of government departments in the early stages of the disaster was not timely enough, while families and communities became the main organizations that temporarily responded to the disaster challenges together. Journalists were not always interested in risk uncertainty, but they were interested in controversy. If the government makes an obvious mistake or if evidence shows that vested interests are trying to advance a position and working to eliminate opposition, the news media will flock to the story. Therefore, the topics of responsibility and criticism received much attention from the media coverage. In addition, meteorological statistics showed the precipitation still exceeded the average of previous years during the 3 days after the flood began, so the disaster’s intensity was likely to keep increasing. As threats continued to grow, it became ever more important for media outlets to gather more knowledge, effectively display and communicate uncertainty, and encourage the public to make good decisions. Survey data show that the official news media gained a higher trust in informing people about changes. However, the limitation was that they only reported or criticized objective reality and did not make significant contributions to effective dialogs or social mobilization actions.

Each request for help and individual contribution was noted with the solution in the same line of the spreadsheet as if the OCD was a list of problems to be solved by getting the attention of the various actors. Even the structural framework of the OCD and institutional specifications were discussed by volunteers and online users on a specific sub-page, and anything appeared negotiable. Government officials were also involved in the communication process and used the reliable information thus made available to support decision-making, such as sending firefighters to safeguard confirmed evacuation routes and distributing food and medical supplies to shelters. One of the emergency management staff told us:

*A portion of the trustworthy information in this OCD was used to discuss and design the measures in an attempt to resolve some of the issues once and for all. However, when talking to colleagues in the opinion monitoring department, they complained that the information on social media such as Weibo was too fragmented and that the process of verifying the timeliness and authenticity of each message hindered communication efficiency. (Interviewee C1)*

Unlike fear appeals or adversarial communications, issue-oriented communications focus on “what is to be achieved” and prioritizes the establishment of a participatory and constructive dialog platform. The OCD’s online users fully exercised their right to participate in public affairs under multiple interactive public discourse channels and reasonably expressed their opinions and affective appeals, which may have generated positive evaluations of their risk communication effectiveness. Therefore, this constructive communication expected the resolution of crisis issues to have the effect of alleviating individual and group fears.

### Controlled Content Credibility

Disinformation can reduce the credibility of media platforms (Bontcheva et al., 2013) and undermine the legitimacy of governments’ risk management policies (Kim et al., 2019). Therefore, validating credibility is a key aspect that affects the effectiveness of risk communication. While journalistic objectivity is derived from the journalist’s individual cognitive framework and the organization’s gatekeeping rules, fact-checking is often carried out after publication. Social media, on the other hand, relies excessively on algorithms to filter information, which appear to be ineffective and have caused a series of ethical controversies in human–computer interaction. The OCD’s credibility was controlled through two layers: affordances about normability and maintainers as gatekeepers. The validation process was that every piece of information related to this flood was verifiable by external credible sources, which rejected the tendency for private matters or false facts to become public affairs. Inappropriate sources were hearsay, open forums, and subjectivist personal observations. Normability was analyzed in Section “Normability” as a potential interaction rule on the online space that all actors must follow. Meanwhile, the gatekeepers came from an active group of volunteers who guaranteed the OCD’s stability through nonautomated operations. On the first day the floods swept through Zhengzhou City, this volunteer team grew quickly from three members to dozens and kept growing in the days that followed. This team verified each geographic location and contact information and assessed the priority of the requirements. A clear division of labor supported efficiency in emergency situations, minimized disinformation, and promoted collective intelligence. Thus, the team demonstrated methods and strategies for fact-checking media information.

## Square for the Affective Public

### Temporary Public Sphere

Online users appeared to discuss the floods as a public affair rationally in the OCD, and some consensus was reached around controversies of public interest, such as how individual donations were used, priorities for rescuing stranded people, and rules for reviewing the credibility of information. Furthermore, technological availability supported actors to expand weak social ties and activated potential actions. Impartial, democratic, and rational consultation is associated with the quality of the decision-making process and can provide legitimacy criteria for the public to gather in digital platforms. The ideal situation about the public sphere and spiritual interactions can be discussed as depicted by Hannah Arendt and Habermas. While academics continue debating whether digital technologies offer potential benefits that influence the process of constructing the public sphere, Dahlgren (2005), for example, argued that most online interactions are apolitical or entertainment oriented, which may limit the deliberative potential of public affairs. However, in reality, the self-awareness and reflection of online user activity through the OCD tended to be rational and even provided a legitimate digital identity. Some users extend their volunteer contributions to adopt management roles, help mediate conflicts, negotiate risk communication procedures, and engage in other public online activities. This analysis suggests that we should use this concept in light of the discursive intent of the public sphere, which is to appeal to some form of rational public consensus that inspires political action and counters authoritarian forms of decision-making (Calhoun, 1992).

Habermas (1989) initially saw the combination of public and private affairs as symptomatic of the collapse of the structure of the public sphere, and he criticized the mass media as the main driving force behind this trend. During the 2021 Henan flood, the boundaries of public affairs tended to blur even more: On the one hand, individual actions and sentiments previously rejected by theoretical values became an essential part of risk communication; on the other hand, networked publics organized themselves around participatory autonomous actions that relied on reciprocal relationships with each other and that aimed to improve society. However, the uncertainty of the boundaries did not mean that the OCD was a free-flowing, open, discursive space. We, therefore, followed classic idea of Dewey and Rogers (1927) of media democracy, which prompted us to attempt to identify the public character of the media through shared problems and solutions. The publicness of the OCD was reflected in a common framework of action constituted by social experience and supported by technological affordances. It unified the concerns of local communities in a political sense and allowed access to external users, which promoted the social mobilization of citizens rather than their representation. Rather than simply expressing opinions through their digital participation, online publics with citizenship used their characteristics to mobilize and act. Regrettably, however, the concept of the public sphere is like a “superconductor” in the sense of physics, where the ideal situation can be maintained only at a specific temperature or pressure. Regarding OCD usage, work pressures force employees to communicate efficiently, and humanitarian crises force the public to organize response actions in an orderly manner. While technological affordances supported an online space and actively fostered the potential of the public sphere, the spirit of publicness was sequestered in the specific scene of risk communication during the flood; thus, it was “temporary.”

### Reconceptualizing the Affect

While the use of the public sphere to conceptualize the online space shaped by OCD faces many limitations, the “affective turn” offers an alternative, sustainable insight and the opportunity to challenge the distinction between private and public (Clough and Halley, 2007; Ekström et al., 2016). Therefore, before discussing RQ3, we must reconceptualize the “affect.” Our interpretation follows understanding of Papacharissi (2015) that affect is a subjective experience of pre-emotive intensity that provides discourse and narrative for networked publics. Furthermore, it implies the potential and urgency for people to act based on their sentiments (Damasio, 1994). Affect supported the OCD users to reflexively reflect on and construct individual narratives based on objective reality, including expressing concerns about public affairs without having to identify to or negotiate political identities with the publics, which could be considered part of sustainable citizenship. Further, identity hierarchy and cultural capital were not the dominant logics of the OCD, but they were profoundly influential in the social media or journalistic field. For example, we found that under the topic of #2021 Henan floods, the opinion expression behavior of online publics on social media platforms was easily influenced by key opinion leaders, mainly in the form of emotional contagion and opinion convergence. Likewise, objectivity rules required journalists to reject subjective interference, but political dictates or commercial interests still controlled news coverage.

Emotions can be viewed as subjective mental activity, but affect is not entirely nonrational. As a relational discursive practice (Döveling et al., 2018), affect marks our original perception of the real world and can support our potential actions. We easily identified affective flows among the OCD users with collective, communal, and political characteristics, such as anxiety and excitement, fear and confidence, and despair and hope. After identifying and labeling the text with affective words through the LIWC-22 dictionary, we found that anxiety was the predominant emotion (*N* = 56.57%), followed by positive (*N* = 21.83%), sad (*N* = 12.66%), and angry (*N* = 7.35%). The dimensions of emotion and examples of each sentiment are reported in Table 2. The online users were also stimulated by the humanitarian crisis to develop deep affective tones of compassion and solidarity, which influenced the potential direction of the affective flow. To be clear, our understanding is based on a “mediated emotion” (Deacon and Stanyer, 2014) or “digital affect cultures” (Döveling et al., 2018).

**Table 2:** The affective flows in online collaborative document.

Dimensions (*N*%)	Examples of each sentiment
Anxious (56.57)	The water and electricity were cut off. The water level was nearly 1.8 m and there was already a companion dead, the situation was urgent!
	My father has been missing for more than 18 h. Anyone nearby please help inquire about his whereabouts.
	The water level was up to the roof and the gas station was leaking oil, putting the place at risk of explosion.
Positive (21.83)	Go for it! The more I watch the news these two days, the more I want to weep! Zhengzhou will be safe and sound ah!
	The power of people’s unity is infinite!!!
Sad (12.66)	My new laptop was damaged, so sad I was!
	Natural disasters are merciless, and we will mourn the victims.
Angry (7.35)	Why did the drainage system fail? Urban planning needs urgent improvement!
	48 h have passed and we have not waited for the firefighters! It seems we have to survive on our own!
Others (1.59)	Thanks to firefighters, I’m safe (Happy).
	The rainstorm will continue and I think the situation will worsen (Negative).

### Digital Affective Space

While text mining and semantic recognition helped us identify the main affective flows and initially answer RQ3a, the analysis of people’s social mobilization around affective ties (RQ3b) requires focusing on the digital space’s structure and tracking users’ footprints. The relational dimension inherently signifies emotion as collective (Gergen, 2009), which indicates that networked publics that are transformed by technological affordances (Boyd, 2010). The OCD’s technological affordances supported the creative connection of distributed individual nodes, incorporating more weak ties and even strangers. Therefore, the OCD could be seen as a surface or space for affective flows (Carroll and Landry, 2010), where information was shared and affect were unfolded. Online users expressed their sentiments through emoji, verbal text, or various colors, and may have been influenced by other comments to take possible actions. Especially with common experiences such as mourning, death, despair, and grief, the related affect could be reinforced during flowing. Those affective footprints showed the intersectionality of intimacy between different individuals as well as groups, commenting on certain shared affective experiences. In addition to risk communication, the affective footprints covered a wide range of topics, including counseling for mental health, contributions to community action, and reflections on urban life.

Moreover, a “Wailing Wall” was established by the OCD’s administrators to pay tribute to the deceased and to provide encouragement to others. As an ongoing open message board, the space allowed all users to share their individual disaster experiences. The interweaving and overlapping of private memories from different perspectives, which collectively created a portrait of life in a catastrophic event, could have contributed in part to the practice of a “democratizing memory” (Walter et al., 2012). Significantly, these affective flows were histories written independently by online users, not journalists or political figures. The OCD was shaped as a distinctive space for experiencing affective flows, conveying the collective and public feelings of disaster victims, volunteers, government officials, and other netizens. Communication within the digital affective space not only revolved around sentiments and emotion but also supported social interactions. In contrast to the material reality space, the affective space could be a combined layer of the real event and permanently retained as a special form of mediated memory, especially in a context of uncertainty and threats.

### Activities of the Affective Publics

Affective expression has always been a cultural practice (McCarthy, 1994). Digital platforms do not directly facilitate the negotiation of collective identities; thus, online users’ affective expression requires specific political and cultural discourses. Concepts such as “public feelings” (Cvetkovich, 2007) and “intimate publics” (Berlant, 2008) imply that previously private individualized affective expressions can be cultural, collective, public and political in mediated spaces. While personal feelings arise from stimuli in the external environment, large-scale affective flows are derived from social events. Public affairs invite and sustain the aggregation and mobilization of networked publics based on affective ties, which shape an “affective public” (Papacharissi, 2015). As Hemmings (2005) argued, social mobilization from the internet is not achieved exclusively by appealing to reason or interests but also by creating “affective dissonance” or “affective solidarity” with considerable potential.

The OCDs’ affective networks were not generated solely by digital collaboration and information-sharing, while we can continue to search and identify the affective publics’ activities by accessing this digital space. Thus, their digital interactions made the affective network visible. Any user can easily identify the digital footprints left by affective publics, especially certain rhythmic affective flow (Papacharissi, 2015). This sense of rhythm was reflected in the symbiosis or interweaving of positive and negative emotions, resulting in secondary or cumulative expressions of action. As Belli and Alonso (2021, p. 6) claimed, this intertwining and conflicting emotional contagion was reflected in the statement, “*People suffer physical and psychological pain, but they know that society will prevail*.” The affective public shaped by OCDs was thus a fusion of specific experiences, histories, cultures, and sensations of a certain moment, a structure that was hyper-local. The religious, regional, and identity differences between the participants were no longer emphasized by the OCDs’ users, who instead focused on the experiential and affective power that connected them. The respondents’ affective experience of participating in online activities on the OCDs was described as “*an atmosphere of intimacy*” (Interviewee A1), “*a game of hope and despair*” (Interviewee A5), and “*full of compassion*” (Interviewee B2). The affective publics’ discourse practices constructed online activities as a humanitarian crisis situation, and temporarily neglected the critique of governments’ failures. Likewise, socialites could not emphasize privileges on the OCDs, such as controlling public opinion or receiving more donations.

Based on the above understanding, then, this paper tends to describe OCDs as a square for affective publics. Compared to “space,” the term “square” has more a public characteristic: It is both a digital and visible place of interactions, capable of being located by external actors, and an idealized deliberative space, allowing citizens to gather and discuss public affairs.

## Conclusion and Discussion

As Livingstone (2009) declared, the era of “the mediation of everything” has arrived because advances in digital technology have supported media in their characterization of modernity and reshaping of social interactions (Plantin et al., 2018). Along with the rapid expansion of digital materiality systems, media have become the infrastructure of modern society. Different from how it was understood in the era of mass media, digital media is no longer simply a channel for information communication but also a platform for organizing and generating social action and supporting public interest and institutional arrangements (Plantin et al., 2018). As Debray (1996) noted, the media is no longer merely a static, material “object,” but is now a dynamic adjustment process with initiative and dynamism. Our answers to the three research questions supported this claim, and the identified sub-processes formed a complex picture of mediatized research. OCDs act as the media logic that functions to establish connections, form interactions, and transform different forces, and they complete the transformation from ideas to material forces through technological and organizational practices, connecting different social groups, forming new interactive relationships, and mediating the allocation of social resources.

### Theoretical Implications

**Figure 1:**
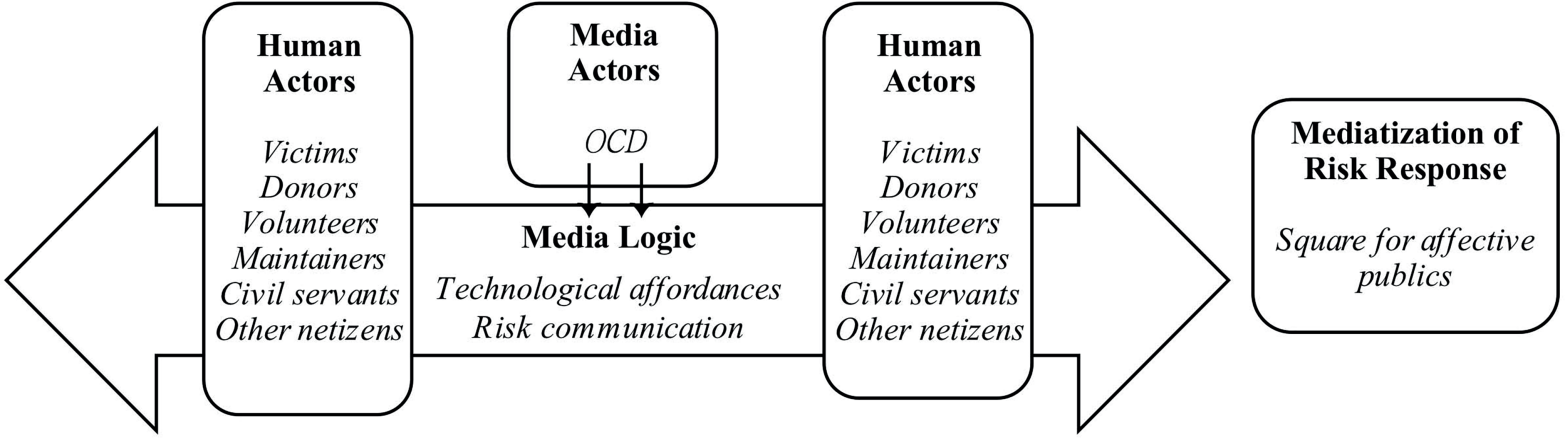
The mediatization of risk response through online collaborative document.

Institutionalism and constructivism are the main research agendas of mediatization studies, with the former focusing on defining media logic (e.g., Shehata and Strömbäck, 2021; Blassnig and Esser, 2022), and the later tending to describe the reconfiguration of social reality in a polymedia environment (e.g., Cui, 2019). This paper first inspired an institutional lens for the mediatization study as a contextualized and integrated formula that covers the process of interconnectedness between media and social life (Ekström et al., 2016; Nowak-Teter, 2019). We discussed the possible forms of media logic and the mediatization of risk response through three empirical sub-processes at the technological and contextual dimensions. These results could be used to refine the mediatization theory model. Figure 1 shows how OCDs reshaped the risk response. The following are the theoretical contributions of this paper in the fields of explaining media logic and describing the mediatization of social interactions.

#### Technological Affordances

This study showed a normative way of using affordances. We found that four dimensions of technological affordances—editability, accessibility, activability, and normability—supported the interactions between OCDs’ functional features and users’ action potential. Some communication studies have focused more on some media’s specific functions and characteristics but have ignored longitudinal changes (Tenenboim-Weinblatt and Neiger, 2018) and perceptual factors such as emotional activities (Nagy and Neff, 2015). In the case of smartphones, for example, researchers have summarized more than 50 types of affordances (Jin and Cai, 2021). This paper, therefore, advocates a more generalized form of technological affordances and emphasizes its theoretical premise, which responds to the call from Evans et al. (2017).

#### Risk Communication

Media are often considered to play an important role in the social amplification of risk (Kilgo et al., 2019), and this study reveals that OCDs can be used strategically by the public to engage in risk response actions because of the technology’s media logic, such as the participatory information release, constructive issue-oriented communication, and controlled information credibility. This information-sharing network was based on practical contributions rather than cultural capital or power confrontation and led to coordination mechanisms that emphasized participation, reciprocity, and reputation. Therefore, as a contextual element and discursive practice of media exposure, risk communication provided the online publics a socialization channel and framework for action. Compared to some functionalist studies of risk communication (Fang et al., 2021), this paper showed the possibility of considering risk response through a contextualized lens, which facilitates the discussion about the extensive social consequences of media exposure behavior. As Wardman (2008) declared, going beyond the normative, instrumental, and substantive imperatives typically employed in the utilization of risk communication requires the consideration of all potential interactions in modern society.

#### Affective Publics

The form of social practice supported by OCDs appeared superficially to be a form of public deliberation, which pointed initially to a discussion of publicness. Academic debate is growing about whether digital media constitute a new public sphere (see Kaluža, 2022; Mpofu et al., 2022), and this study provides a case in a risk context. We attempted to claim that shaping the affective publics was one of the sustainability implications of OCDs. As a unique space for experiencing psychological activities, OCDs preserved the communal sentiments of the disaster victims, volunteers, government officials, and other netizens during the flood. The interweaving of affective flows such as anxiety and encouragement allowed social mobilization within OCDs to de-emphasize cultural, religious, regional, and identity differences among the participants. Dominated by the media logic, the discursive practices of the affective public constructed the online activity as a humanitarian crisis situation and temporarily neglected critical reflection on government failures. In particular, the mediatization of death, mourning, and grief can led to enhanced and diffused affective flows and give rise to possible individual narratives (Shi, 2022). Compared to disaster coverage, which is often controlled by journalism, the affective public achieved a degree of subjectivity in the form of writing media memories and leaving affective footprints, replying to studies of Burgess et al. (2018) and Lagerkvist and Andersson (2017).

Social action is becoming increasingly dependent on media logic (Nowak-Teter, 2019), especially the hybrid media systems of the digital age (Özkula, 2021). These sub-processes together provide a complex picture to capture the broad consequences of media in times of emergency. In this sense, we tend to see mediatization as an approach (Couldry and Hepp, 2013; Ekström et al., 2016). An open research agenda aims to understand how media reshape social practice; thus, it discusses broader meanings and sociocultural shifts rather than continually highlighting the media’s characteristics. We thus explain the institutional significance of mediatization theory by focusing on the media logic that guides organizational activities, prescribes typical behavior, and reveals potential consequences. In addition, we attempt to affirm that in a post-pandemic era replete with risk and uncertainty, humans can still creatively adapt technological conditions to achieve their goals. The insistence on human subjectivity facilitates a profound understanding of the nature of technology and affect, and thus the values of this paper lean toward humanism. We are pursuing a hyper-local discussion, and the social context is not limited to Mainland China. OCD products from Google and Microsoft are also widely used in North America and Europe, and affective publics are equally active on Facebook or Twitter. We look forward to receiving critiques and responses from the academic community.

### Practical Implications

Online collaborative documents aim to provide a response method for online social mobilization in a high-stress environment. As the 2011 Queensland floods have shown (Bunce et al., 2012), when government response is temporarily stymied, the public can perceive environmental changes and organize mutual aid actions through digital platforms. In the technological affordances section, we confirmed that OCDs perform well in a crisis compared to other digital technology systems, especially a participatory, dynamic, and constructive risk communication and information-sharing network. This research can inform government departments and organizations as they develop their digital response strategies so they can provide relevant and accessible information to the public in the event of a natural disaster. OCD technology achieves the immediacy and effectiveness of risk response. In addition, the use of an OCD in the 2021 Henan flood encouraged people who may be deeply at risk thereafter to actively participate in response actions. For example, with the Omicron virus pandemic on Mainland China in early 2022, successive community lockdowns were implemented in various cities. However, instead of being depressed, the concerned public actively used OCDs to support self-organized disaster relief. We can easily find the footprints of these risk communication and emotional flows in social media from places such as Shanghai^1^ and Jilin City.^2^ This situation seems to echo the California wildfire case (Sutton et al., 2008), where, instead of waiting for official arrangements, the affected groups first took steps on their own to remain physically and mentally healthy.

### Limitations and Directions for Future Research

The flood disaster had already been resolved by the time we conducted the study, and data collection relied on participant recall and self-assessment, which may have overstated or underestimated the true study sample. Furthermore, while the findings to date are far from conclusive, they do respond to many of the previous findings and suggest a range of alternative hypotheses. Future work may add more quantitative elements and insights, such as examining how maintainers’ daily practices affect the rules of OCDs as digital space, because it is a challenging issue for maintainers to take the necessary actions to deal with the influx of advertisers, pranksters, and unreliable sources. In addition, the “Wailing Wall” shows that OCDs can store online users’ affective expressions, so tracking respondents’ psychological fluctuations during risky events will be required. We also think it is necessary to give focus on the discursive rhetoric of technology companies such as Google and Tencent, as a growing number of OCD products claim risk response as their primary goal among the pandemic.

## Data Availability Statement

The original contributions presented in the study are included in the article/Supplementary Material, further inquiries can be directed to the corresponding author.

## Ethics Statement

The studies involving human participants were reviewed and approved by Institute of Journalism and Communication, Sichuan Academy of Social Sciences. The patients/participants provided their written informed consent to participate in this study.

## Author Contributions

HJ contributed to the conceptualization, literature review, and manuscript writing. WG performed the questionnaire development and contacting participants. YZ performed the data analysis. WC conducted interviews. JP performed the supervision and financial support. All authors contributed to the article and approved the submitted version.

## Funding

This work was supported by The National Social Science Fund of China (21XXW003), The Provincial Philosophy and Social Science Program of Sichuan (SC21B077), and The Sixth Academic Innovation Program of the Graduate School of Sichuan Academy of Social Sciences (HJ).

## Conflict of Interest

The authors declare that the research was conducted in the absence of any commercial or financial relationships that could be construed as a potential conflict of interest.

## Publisher’s Note

All claims expressed in this article are solely those of the authors and do not necessarily represent those of their affiliated organizations, or those of the publisher, the editors and the reviewers. Any product that may be evaluated in this article, or claim that may be made by its manufacturer, is not guaranteed or endorsed by the publisher.

## Footnotes

^1^To view the original link, please click https://docs.qq.com/sheet/DRUdjRmttZmtrWGVq?u=71745445c17f41b9bdaa1dcaba6d5968&tab=t2pjr3&scode=&code= 081Wiq000LmEMN1f4B000dgESn0Wiq0q&state=onweixinlogin&_t=1651667325209.

^2^To view the original link, please click https://docs.qq.com/sheet/DZmFnbG9ubVh0Zkpz?tab=iux1qx&u=80432a04236d4d75bff9d6fbfea6e915.
