# Supplementary material for: Online Collaborative Documents as Media Logic: The Mediatization of Risk Response in the Post-pandemic Era
Source: Front Psychol. 2022 Jun 23;13:892569. doi: 10.3389/fpsyg.2022.892569 (PMC9260903; doi:10.3389/fpsyg.2022.892569)
Supplement: Supplementary file 1 [file Data_Sheet_1.docx]

Appendix

# **Measurement Items**

The measurement items were derived from the questionnaire and the relevant data were mainly applied to the discussion of RQ2. **Table 1** reports participants’ demographic characteristics.

Table 1. Demographic characteristics of the respondents (N=420).

|  | | N | % |
| --- | --- | --- | --- |
| Age | 18-28 years | 252 | 60.0 |
|  | 29-50 years | 164 | 39.0 |
|  | Above 50 years | 4 | 1.0 |
| Gender | Male | 180 | 42.9 |
|  | Female | 240 | 57.1 |
| Education | Secondary school or below | 4 | 1.0 |
|  | High school | 16 | 3.8 |
|  | College | 116 | 27.6 |
|  | University or above | 284 | 67.6 |

**1.1 Frequency of media use.**

Participants were asked to report their frequencies of using traditional media and social media. Questions were presented on a Likert scale (1 = never, 5 = always). For traditional media, we measured official news media and commercial news media. Referring to Wu (2021) et al, People’s Daily, CCTV, Xinhua News Agency, and local media were included in the category of official news media. In addition, we asked respondents to estimate and report the frequency of their exposure to commercial news media such as The Paper, The Beijing News, and The Nanfang Metropolis Daily. For social media, instant messaging media and information aggregation media were measured. Similar to Facebook, WeChat and QQ provides private communication and personal photos sharing services. Similar to Twitter and YouTube, various forms of information flow are presented on media platforms such as Weibo, Tik-Tok, and Bilibili.

**1.2 Motivation for media exposure.**

Participants were asked to report the priority of their media use motivations, with questions adapted from a study by Lev-on (2012) et al. The five items of environmental perception, help-seeking, charitable donations, psychological adjustment, and surveillance by public opinion were measured by the Likert scale (1 = hardly needed, 5 = completely needed).

**1.3 Media trust.**

Media trust was measured by a five-point Likert scale (1=completely distrustful, 5=completely trustful). We asked participants to report their level of trust in information from official news media, commercial news media, instant messaging media, information aggregation media, and OCD.

**1.4 Data Analysis**

Scale data were subjected to general descriptive and factorial correlation analyses by the researchers through SPSS 24.0 software to explore the online users’ specific activities through OCD in a polymedia environment. The relationship between media use motivation and media trust was tested by Spearman’s Order Correlation Coefficient (SROCC). SROCC is considered to be the superior nonlinear correlator with excellent performance in measuring the order correlation between elements, as demonstrated by several previous psychological and media studies (e.g., Miller and Ulrich, 2001; Tomotake et al., 2003; Buller et al., 2008). These analyses were exploratory in nature and did not acquire statistical knowledge, so we tried to avoid relying on numbers to obtain conclusions. Notably, based on a pre-test (N=50), we found that participants’ mental health indicators such as anxiety and stress were positively correlated with the level of disaster. However, we believe that stratified sampling based on the degree of damage is very difficult and that having large numbers of participants recall and code their distress is also contrary to research ethics. Thus, the analysis of the interview data supported the discussion of OCD-mediated risk communication.

# Results

**2.1 Media exposure**

Table 2. Frequency of media use (N=420).

| Items (range 1-5) | Always |  | M (SD) | |
| --- | --- | --- | --- | --- |
|  | N | % |  | |
| Official news media | 156 | 37.1 | 3.79 (1.212) |  |
| Commercial news media | 56 | 13.3 | 2.93 (1.289) |  |
| Instant messaging media | 268 | 63.8 | 4.5 (0.784) |  |
| Information aggregation media | 228 | 54.3 | 4.21 (1.088) |  |

*M, mean. SD, standard deviation.*

**Table 2** shows the frequency of people’s use of different media platforms. The percentage of participants who rated a specific media outlets as "always use" was counted by us. Overall, instant messaging social media such as WeChat and QQ received the highest frequency of use, with approximately 63.8% of participants reporting “frequent use”, (M=4.5, SD=0.784). Followed by information aggregation social media such as Weibo, Tik Tok, and Bilibili (M=4.21, SD=1.088). Among the four types of media outlets, commercial news media were the least frequently used (M=2.93, SD=1.289).

Table 3. Motivation for media exposure (N=420).

| Items (range 1-5) | Completely needed | | M (SD) | |
| --- | --- | --- | --- | --- |
|  | N | % |  | |
| Environmental perception | 246 | 58.6 | 4.44 (0.788) |  |
| Help-seeking | 168 | 40.0 | 3.83 (1.190) |  |
| Charitable donations | 162 | 38.6 | 3.86 (1.147) |  |
| Psychological adjustment | 142 | 33.8 | 3.71 (1.235) |  |
| Surveillance by public opinion | 156 | 37.1 | 3.80 (1.168) |  |

People’s motivations for media exposure are reported in **Table 3**. Among the five main types of needs, environmental perception received the highest need, with approximately 58.6% of participants indicating a “strong need” (M=4.44, SD=0.788). This was followed by help-seeking (M=3.83, SD=1.19) and charitable donations (M=3.86, SD=1.147). The level of people’s need for psychological adjustment was relatively low (M=3.71, SD=1.235).

Table 4. Participants’ interactions with OCD (N=420).

| Questions | Yes | |
| --- | --- | --- |
|  | N | % |
| Contact | 308 | 73.3 |
| Sharing | 222 | 52.9 |
| Seeking help | 54 | 12.9 |
| Assisting with maintenance | 98 | 23.3 |
| Encouraging others | 182 | 43.3 |
| Providing relief resources | 108 | 25.7 |
| Caring about the progress of rescue | 246 | 58.6 |

**Table 4** reports the participants’ interactions with this OCD. Because the sampling for this study was dependent on the social networks of the researchers, rather than tracking the contact information left by users at the OCD (and that would be contrary to ethical rules), it was essential to ask them to report their exposure to the OCD. A set of questions was used to obtain people’s knowledge and involvement in OCD, which facilitated the researcher to check the quality of the sample and design follow-up targeted interview questions.

2**.2 Media trust**

Table 5. Participants’ trust in particular media. (N=420).

| Items (range 1-5) | Completely trustful | | M (SD) | |
| --- | --- | --- | --- | --- |
|  | N | % |  | |
| Official news media | 278 | 66.2 | 4.52 (0.802) |  |
| Commercial news media | 100 | 23.8 | 3.54 (1.178) |  |
| Instant messaging media | 118 | 28.1 | 3.77 (1.014) |  |
| Information aggregation media | 112 | 26.7 | 3.67 (1.068) |  |
| OCD | 142 | 33.8 | 3.91 (0.996) |  |

**Table 5** reveals the level of people’s trust in particular media. 66.2% of respondents rated information from the official news media as “completely trustful” (M=4.52, SD=0.802), thus it received the highest level of trust. The commercial news media was the least trusted media platform in comparison (M=3.54, SD=1.178).

Table 6. Association between motivation for media exposure and media trust. (N=420).

|  | | Media trust | | | | |
| --- | --- | --- | --- | --- | --- | --- |
|  | | Official news media | Commercial news media | Instant messaging media | Information aggregation media | OCD |
| Motivation for media exposure | Environmental perception | **.387**(.000)** | -.005(.473) | .199**(.002) | .138*(.023) | .215**(.001) |
|  | Help-seeking | .030(.332) | .187**(.003) | .248**(.000) | .244**(.000) | **.301**(.000)** |
|  | Charitable donations | .123*(.123) | .194**(.002) | .228**(.000) | .223**(.001) | **.251**(.000)** |
|  | Psychological adjustment | .187**(.003) | .252**(.000) | **.310**(.000)** | .239**(.000) | .213**(.001) |
|  | Surveillance by public opinion | .230**(.000) | .244**(.000) | .243**(.000) | .191**(.003) | **.253**(.000)** |

*Spearman’s rho correlation coefficient (ρ) was used for correlation calculations. **p < 0.01, *p< 0.1.*

The correlation test between the different subcomponents of media exposure motivation and media trust is shown in **Table 6**. Based on the theoretical perspectives of use and satisfaction and media constructivism, we can explore which media platforms gained trust by satisfying which needs of users during the flood. In other words, their correlation tests provided a preliminary picture of the role of the media in risk communication. The data reported that official news media received the highest trust in satisfying users’ environmental perception needs (ρ = 0.387, p < 0.01), revealing that people prefer to obtain authoritative information from official media outlets during a crisis. While instant messaging social media played the most important role in regulating mental health (ρ = 0.31, p < 0.01), indicating that social media is then used more to feel and express emotions. It is worth highlighting the correlation between media trust in OCD and the need to seek help (ρ=0.301, p<0.01), charitable donations (ρ=0.251, p<0.01), and surveillance by public opinion (ρ=0.253, p<0.01). The above results suggested several possible directions for the role of OCD as a mediator in risk communication practice.

# Main questions for discussion in the focus group

- Can you tell me about your experiences with using media during the 2021 Henan Floods?
- What sorts of things did you use to learn about the floods?
- Can you tell me which media platform you think played the greatest role in the flood or which media platform you tend to trust more? Can you give some reasons?
- Did you have any exposure to OCD prior to this flood? If so, can you describe your contact experience specifically?
- Which media platform were you accessing OCD from?
- Why did you use OCD?
- At what point did you decide to engage with OCD?
- Can you tell me how you participated?
- Can you tell me what you did with the information you found?
- Can you tell me about your thoughts or experiences when you used OCD?
- Can you tell me about technical troubles you've had using OCD?
- Can you tell me about how your understanding of the flood crisis changed (if at all) as a result of your experiences with using OCD?
- Can you tell me about your affective experience with OCD during the recent flood?

# Personalized secondary questions

## For volunteered maintainers (B1-B3)

- Why did you choose to become a volunteer?
- Can you tell me the main tasks of a maintainer?
- Has the manager community discussed changing to another media platform?

## For official government (C1)

- What do you think of OCD-mediated mutual aid rescue actions?
- What role did the OCD’s information play in governments’ risk response?
- Have you considered using OCD for some aspect of risk management in the future?

## For help seekers (A1-A6)

- After posting a request for help on OCD, how often did you receive reply?
- Did OCD help you succeed in getting rid of the threat? If so, can you tell me how you feel after being successfully rescued?
- Which part of the rescue operation do you think OCD helped the most?

## References

Wu, Y., and Shen, F. (2021). Exploring the impacts of media use and media trust on health behaviors during the COVID-19 pandemic in China. *J. Health Psychol.* 27: 1359105321995964. doi: 10.1177/1359105321995964

Lev-On, A. (2012). Communication, community, crisis: Mapping uses and gratifications in the contemporary media environment. *New Media Soc.* 14, 98-116. doi: 10.1177/1461444811410401

Miller, J. and Ulrich, R. (2001). On the analysis of psychometric functions: The Spearman-Kärber method. *Percept. Psychophys.* 63, 1399-1420. doi: 10.3758/BF03194551

Tomotake, M., Harada, T., Ishimoto, Y., Tanioka, T., and Ohmori, T. (2003). Temperament, character, and eating attitudes in Japanese college women. *Psychol. Rep.* 92, 1162-1168. doi: 10.2466/pr0.2003.92.3c.1162
